# Adoption and Practice Variability of Prostate Stereotactic Body Radiotherapy (SBRT) in Latin America: The Role of Continuous Education in Advancing Quality and Standardization

**DOI:** 10.7759/cureus.105380

**Published:** 2026-03-17

**Authors:** Pablo Castro Pena, Lourdes M Suarez Villasmil, Patrick Kupelian, Miguel Gonzalez, Cleverson Lopes

**Affiliations:** 1 Radiation Oncology, CETAC Juncal (Centro de Tratamiento de Alta Complejidad), Buenos Aires, ARG; 2 Faculty of Exact, Physical and Natural Sciences (FCEFyN), National University of Córdoba, Cordoba, ARG; 3 Radiation Oncology, Varian, A Siemens Healthineers Company, Los Angeles, USA

**Keywords:** global health, hypo-rt-pc, latin america, longitudinal training, medical education, pace-b, radiotherapy, rtog-0938, sbrt, stereotactic body radiotherapy

## Abstract

Background

Prostate cancer (PCa) is a highly prevalent disease in Latin America (LATAM), where access to radiotherapy is limited. Stereotactic body radiotherapy (SBRT) is an effective, safe, lower-cost, and more accessible option; however, its implementation requires protocol standardization and continuous education. This study aims to analyze the practices and procedures of a group of LATAM professionals who use the homogeneous Halcyon^TM^ technological platform to deliver prostate SBRT and to determine their concordance with the randomized trials PACE-B (Prostate Advances in Comparative Evidence - SBRT versus conventional or moderately hypofractionated radiotherapy), HYPO-RT-PC (Hypofractionated Radiotherapy for Prostate Cancer trial), and RTOG-0938 (Radiation Therapy Oncology Group trial 0938).

Methodology

A survey was conducted among 37 professionals (radiation oncologists and medical physicists) from 28 institutions in 10 Latin American countries. Concordance of their practices was analyzed across nine variables (five direct: prescription parameters; four indirect: safety and quality procedures) derived from the three reference trials.

Results

High adoption of hypofractionation (HRT) (86.5%) and SBRT (59.5%) was observed. Concordance with the trials was higher for indirect factors (65.0%) than for direct factors (41.3%), with only 36.2% mean concordance with the prescription parameters of the PACE-B trial. In addition, 40.5% of participants did not use SBRT, with lack of experience being the main barrier (66.7%).

Conclusions

Prostate SBRT is progressively consolidating in LATAM; however, meaningful variability persists in both dose prescription parameters and safety and quality procedures, despite access to homogeneous advanced technology. These findings indicate that while access to advanced technology is of critical importance, technology availability alone is not sufficient to ensure standardized, high-quality clinical practice. Strengthening continuous education, protocol harmonization, and robust quality assurance strategies represents a strategic opportunity to enhance consistency, safety, and clinical benefit across the region.

## Introduction

Prostate cancer (PCa) is the second most common type of cancer worldwide, the second most frequently diagnosed malignancy in men [1], and one of the five most common malignant tumors in Latin America (LATAM) [2,3]. In this highly prevalent and high-mortality disease, radiotherapy (RT) has emerged as an effective primary, adjuvant, or palliative treatment option [4,5].

In low- and middle-income countries (LMICs), where cancer incidence is high [2], only 50% of patients eligible to receive RT have access to this treatment [6,7]. This situation is even more critical in low-income countries (LICs), where less than 10% of the population has access to RT services [6,7]. Limitations in access to RT in LATAM are related to deficiencies in regional healthcare systems, such as geographic disparities in the distribution of healthcare centers, predominantly concentrated in urban areas [3,8], and the high dependence on equipment that has been in use for more than two decades [2]. As a result, cancer survival rates in the region are markedly lower than those observed in high-income countries such as the United States or England [9].

Moderate hypofractionation (HRT), with 2.5-3 Gy per fraction, and extreme HRT (>5 Gy per fraction) have been shown to be effective for the treatment of PCa without increasing toxicity compared with conventional fractionation [10-13]. Stereotactic body radiotherapy (SBRT) is an effective and safe treatment modality for patients with localized primary PCa [11,14], delivered in two to seven fractions [12,15], and supported by evidence demonstrating biochemical control, progression-free survival, and toxicity outcomes comparable to those of hypo-fractionated regimens [10-12].

Early phase III evidence from the PACE-B trial (Prostate Advances in Comparative Evidence - SBRT versus conventional or moderately hypofractionated radiotherapy) demonstrated that SBRT delivered in five fractions was associated with low rates of acute and late toxicity, establishing its safety profile in patients with localized PCa. These results supported the initial clinical adoption of prostate SBRT as an alternative to conventional and moderately hypo-fractionated RT regimens [11].

More recently, the mature results of the PACE-B trial confirmed the non-inferiority of SBRT compared with conventional and moderately hypo-fractionated RT in terms of freedom from biochemical or clinical failure, consolidating SBRT as a standard curative treatment option for localized PCa [12].

Shorter treatment courses increase the likelihood that patients will complete therapy, particularly when facing challenges such as limited mobility, economic constraints, or geographic barriers to accessing specialized medical centers. In addition, SBRT is associated with lower costs for healthcare systems [3], reduced staffing demands, and shorter waiting lists [10,16,17], making it an efficient and economically viable option for resource-limited regions with clear patient-centered benefits.

Continuous education of healthcare professionals in LATAM is crucial for the implementation of these treatment approaches [9]. In a prospective cohort study evaluating the implementation of a remote (telehealth) training program in SBRT and stereotactic radiosurgery (SRS) for radiation oncologists, medical physicists, and residents in Peru and Colombia, the effectiveness of longitudinal remote training was demonstrated in promoting the adoption of these techniques and improving professional competencies in LMICs [18].

Despite significant investments in technological advances for SBRT, its adoption has been slow due to limited experience among healthcare professionals with standardized protocols required to ensure safe and effective SBRT delivery with reliable and reproducible outcomes [18]. Radiation oncology departments worldwide offer training opportunities for professionals from LMICs; however, access to these programs is costly and limited [3,8] and contributes to “brain drain,” as trained specialists often remain in high-income countries rather than returning to their home communities [9]. In this context, distance education (telehealth) facilitates continuous education without the need for travel or disruption of routine institutional activities.

Given this backdrop, collaborative regional initiatives focused on protocol harmonization and longitudinal training have emerged as a structured response to these challenges. The Halcyon User Group - Clinical Working Group (HUG_CWG), created and supported by Varian Medical Systems - A Siemens Healthineers Company, was designed as a collaborative education and quality-improvement framework. Through this program, institutions sharing a homogeneous technological ecosystem can exchange clinical experience, align their practices with international evidence, and facilitate the safe and standardized adoption of advanced techniques such as prostate SBRT across heterogeneous healthcare systems.

Participant selection

The sample was drawn from a population of Halcyon^TM^ users from Varian Medical Systems, an O-ring linear accelerator designed for 100% IGRT delivery, integrated with the ARIA® oncology information system and Eclipse® Treatment Planning System from Varian Medical Systems - A Siemens Healthineers Company (Palo Alto, CA) (Varian Ecosystem) in LATAM. This study, supported by Varian Medical Systems - A Siemens Healthineers Company, aimed to assess the state of the art of prostate SBRT in the region.

The selection of this population was also aligned with the HUG_CWG framework, conceived as a collaborative environment for continuous education and quality improvement. The data generated by this survey were intended not only to characterize current clinical practice but also to inform the design, prioritization, and early implementation of educational interventions within the HUG_CWG program, including structured academic activities such as webinars.

The decision to include a population operating within a homogeneous technological platform (Varian Ecosystem) was intended to minimize variability related to heterogeneous RT technologies, thereby reducing dispersion in treatment quality and isolating clinical practice patterns as the primary source of variability in the analysis.

The Halcyon system is designed to be transported in a shipping container, installed within two weeks, be affordable and easy to maintain in LMICs, and to quadruple the number of patients treated while maintaining the same RT quality as the most advanced treatments delivered in the United States [7]. Halcyon technology (O-ring LINAC) has been validated for the delivery of radiation treatments, including SBRT, with performance equivalent to that of conventional C-arm LINAC systems [19-22].

Objectives of the study

The primary objective of this study was to describe current clinical practices in the implementation of prostate SBRT among radiation oncology professionals in LATAM operating within a homogeneous technological ecosystem.

The secondary objective was to evaluate the concordance between these clinical practices and the protocols reported in major randomized trials supporting prostate SBRT, specifically PACE-B, HYPO-RT-PC (Hypofractionated Radiotherapy for Prostate Cancer trial), and RTOG-0938 (Radiation Therapy Oncology Group trial 0938).

## Materials and methods

Sample selection

A non-probabilistic convenience sample of 37 professionals (radiation oncologists and medical physicists) using Halcyon^TM^ technology was selected from 28 institutions across 10 Latin American countries. Participants were identified through the Halcyon User Group - Clinical Working Group (HUG_CWG) network, which includes institutions using a homogeneous technological ecosystem. The survey invitation was distributed electronically to professionals within this network between March and July 2024.

All participants provided informed consent for the use of anonymized data and signed a confidentiality agreement regarding the information shared.

Statistical analysis

Each professional completed a 24-item questionnaire hosted on the Microsoft Forms® platform (Microsoft Corp., USA). The survey was administered in the participants' local language (Spanish or Portuguese) and collected digital information on professional role, institution, country of practice, and clinical practices related to the use of radiotherapy for PCa. Responses were compiled into a spreadsheet, from which absolute frequencies (number of responses) and relative frequencies (percentages) were calculated using JASP software version 0.19.2.0 (https://jasp-stats.org/).

Among the survey questions (Appendix), nine key variables related to prostate SBRT practice were selected: 1) prescription dose in patients with Gleason Score (GS) 6 (3+3) or 7 (3+4); 2) prescription dose in patients with GS 7 (4+3) or 8 (4+4); 3) prescription of dose to the planning target volume (PTV) equal to or lower than that to the clinical target volume (CTV); 4) number of fractions delivered; 5) the inter-fraction spacing schedule of SBRT; 6) assignment of a urethral dose constraint; 7) use of magnetic resonance imaging (MRI) for prostate target delineation; 8) bladder and rectal preparation using specific dietary protocols; and 9) use of intraprostatic fiducial markers.

The questionnaire was developed by the study investigators based on clinical variables derived from published randomized trials and SBRT clinical protocols. Prior to distribution, the survey was reviewed by a multidisciplinary group of radiation oncologists and medical physicists to ensure clarity and clinical relevance.

The PACE-B trial [11,12] was selected as the primary reference study, as it represents the only phase III randomized trial evaluating prostate SBRT delivered in five fractions with mature oncological outcomes. This trial was therefore used as the principal benchmark for assessing concordance with direct prescription parameters.

The HYPO-RT-PC [23] and RTOG-0938 [24] trials were included as complementary reference studies, as they provide relevant guidance on treatment delivery schedules, image guidance, target delineation, and safety and quality assurance procedures. While these trials do not represent the primary phase III evidence for five-fraction prostate SBRT, they were considered essential for evaluating indirect factors related to treatment accuracy, reproducibility, and patient safety.

Concordance between individual clinical practices and the protocols described in the randomized trials (PACE-B, HYPO-RT-PC, and RTOG-0938) was calculated by comparing each participant’s responses with predefined variables extracted from the trials. For each variable, adherence to the protocol recommendation was scored as concordant. The percentage of concordance for each participant was then calculated based on the proportion of variables aligned with each trial protocol. A participant who followed all recommendations of a given trial was assigned 100% concordance for that trial. Because some variables were shared across trials, adherence to a given recommendation could increase concordance with more than one trial simultaneously. All calculations were performed using the Pandas library in Python version 3.8 (https://www.python.org/).

## Results

Participation

**Figure 1:**
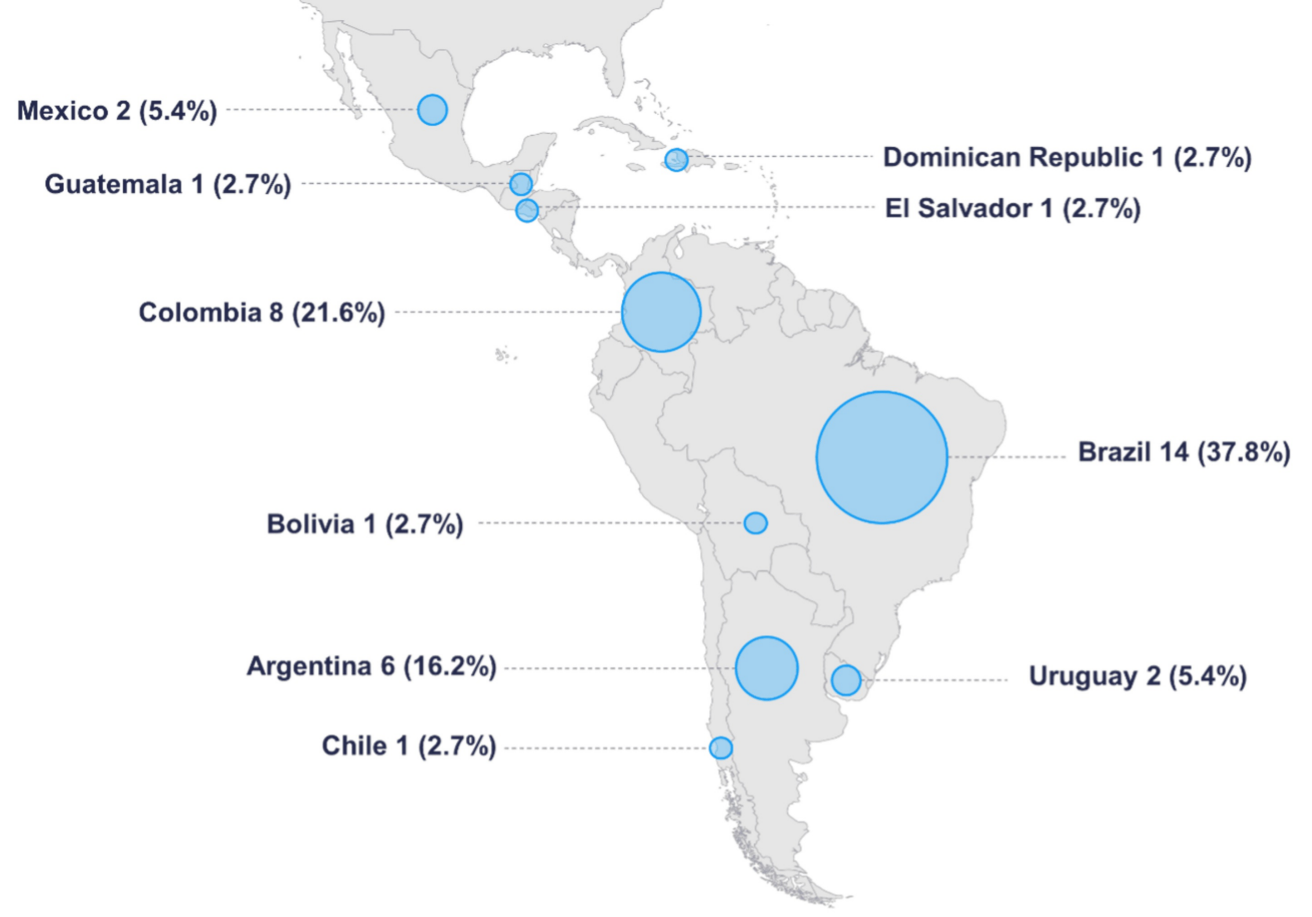
Country of practice of the participants. Image Credits: Pablo Castro-Peña, Lourdes Suárez-Villasmil, Patric Kupelian, Miguel Gonzalez, and Cleverson Lopes. The SVG image used for the map was freely taken from Wikipedia [25] and adapted by the authors of this work using Inkscape software (https://es.wikipedia.org/wiki/Archivo:Latin_America_regions.svg).

Thirty-seven professionals from 10 Latin American countries participated in the study, with Brazil, Colombia, and Argentina being the most represented (Figure 1).

Use of the technique

**Figure 2:**
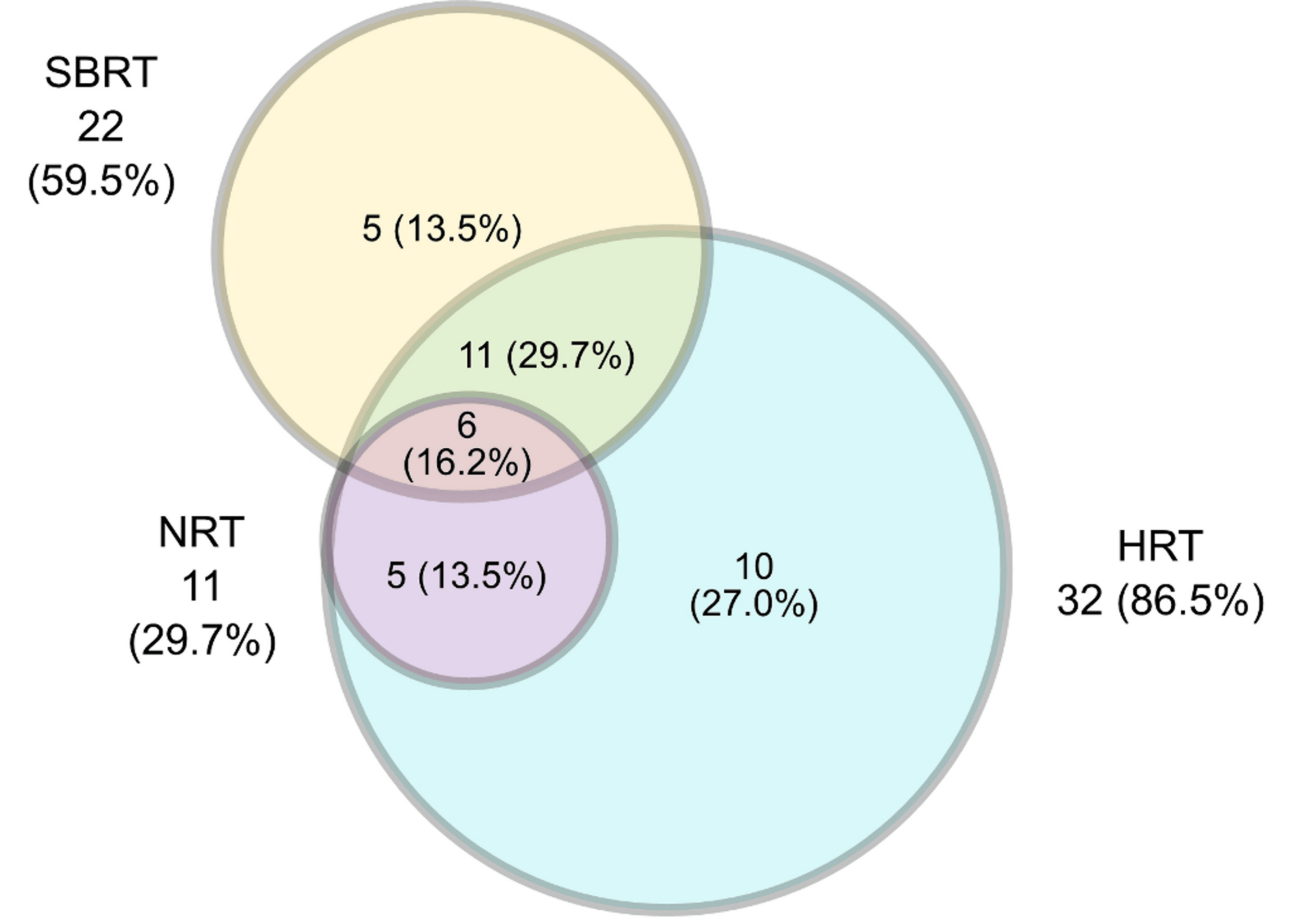
Representation of the number of professionals (percentage in parentheses) who perform moderate hypofractionated radiotherapy (HRT), stereotactic body radiotherapy (SBRT), and normofractionated radiotherapy (NRT), individually or in combination, by institution. Image credits: Pablo Castro-Peña, Lourdes Suárez-Villasmil, Patric Kupelian, Miguel Gonzalez, and Cleverson Lopes (generated with Microsoft PowerPoint (Microsoft Corp., USA))

The most frequently used prostate radiotherapy techniques were hypofractionation, reported by 32 (86.5%) respondents, and SBRT by 22 (59.5%) respondents, applied either individually, in combination with each other, or in association with normo-fractionation, which was still used by 11 (29.7%) of professionals but never as an exclusive modality. As shown in Figure 2, all three RT modalities are applied within the same institution according to institution-specific criteria. No respondent reported the use of normo-fractionated radiotherapy (NRT) as the sole modality for prostate radiotherapy.

At the time of the survey, 15 (40.5%) participants were not using SBRT for the treatment of PCa. Among the 22 (59.5%) participants who did use SBRT, 11 (50.0%) had applied it in no more than 50 patients, one (4.5%) in 51-100 patients, and six (27.3%) in more than 100 patients. Among those who did not perform prostate SBRT, five (33.3%) were in the process of implementation, while 10 (66.7%) did not use the technique due to a lack of experience.

Practices and randomized trials in SBRT

The practices and protocols implemented by professionals performing prostate SBRT, as reported in the survey, are presented in Table 1. Within the SBRT group, all participants applied a specific bladder and rectal preparation protocol, as described in the PACE-B study (Table 2). Treatment was delivered in five fractions by 20 (90.9%) respondents, in accordance with PACE-B, and 19 (86.4%) used magnetic resonance imaging (MRI) to delineate the prostate volume, consistent with imaging recommendations reported in the HYPO-RT-PC and PACE-B protocols. A total of 15 participants (68.2%) reported obtaining dose constraints for organs at risk (particularly rectum and bladder) from published randomized clinical trials.

**Table 1:** Practices and protocols in the delivery of stereotactic body radiotherapy (SBRT) for prostate cancer (PCa) (n = 22).

Practices and protocols	Frequency, n (%)
Have a specific bladder and rectal preparation protocol	22 (100.0%)
Perform SBRT in 5 fractions	20 (90.9%)
Use MRI to delineate the prostate volume	19 (86.4%)
Perform SBRT on consecutive days	18 (81.8%)
Assign a dose limit to the urethra to be respected	18 (81.8%)
Use of SBRT in patients with prostate recurrence	17 (77.3%)
Use inter-fraction IGRT (prior to SBRT initiation only)	16 (72.7%)
Obtain dose limits for organs at risk from published randomized trials	15 (68.2%)
Prescribe the same dose to the PTV as to the CTV for the primary prostate	14 (63.6%)
Use rectal enema	14 (63.6%)
Perform an SIB in the prostate (higher dose than prescribed to the prostate CTV) if a tumor nodule is identified by MRI	13 (59.1%)
Use of SBRT in post-prostatectomy patients	11 (50.0%)
Perform prophylactic SBRT in pelvic nodal areas for primary PC patients (25 Gy in 5 fractions)	7 (31.8%)
Routinely use intra-prostatic fiducials for SBRT delivery	3 (13.6%)
Assign a dose limit to the bladder trigone to be respected	3 (13.6%)

**Table 2:** Specifications of the published randomized protocols. Variables marked with * were considered in the concordance analysis between participants and the three published randomized trials. PACE-B: Prostate Advances in Comparative Evidence – SBRT versus conventional or moderately hypofractionated radiotherapy, HYPO-RT-PC: Hypofractionated Radiotherapy for Prostate Cancer trial, RTOG 0938: Radiation Therapy Oncology Group trial 0938

	PACE-B	HYPO-RT-PC	RTOG 0938
Reference	Tree et al., 2022 [11] Van As et al., 2024 [12]	Widmark et al., 2019 [23]	Lukka et al., 2018 [24]
Phase	III	III	II
N	874	1200	255
Fractions*	5	7	5
Frequency*	Every other day, Daily	Every other day, Interdaily	Every two days
α/β Prostate	3	3	3
Gy Prostate PTV*	36.25	42.7	36.25
BED/EQD2 PTV	123.8 / 74.3	129.5 / 77.7	123.8 / 74.3
Gy Prostate CTV*	40	42.7	36.25
BED/EQD2 CTV	146.6 / 88	129.5 / 77.7	123.8 / 74.3
PTV Prostate (mm)			
Posterior	3-5	7	3
Rest of	4-5	7	5
Technique	IMRT / VMAT	3D / IMRT	IMRT / VMAT / Protons
Stage			
T	T1c–T2x	T1c−T2 vs T3a	cT1-2a
N	0	0	0
M	0	0	0
Gleason score*	7 (3+4)	2-6 vs 8-10	2-6
PSA (ng/ml)	< 20	< 20	< 10
MRI to contour volume*	Highly recommended	Recommended	Does not refer to
Urethra doses *	Yes	No	Yes
Intraprostatic fiducials*	Recommended	Recommended	Does not refer to
Bladder and digestive preparation*	Recommended	Does not refer to	Does not refer to

A total of 18 (81.8%) professionals reported delivering SBRT every other day, in line with the recommendations of HYPO-RT-PC and PACE-B. Similarly, 18 (81.8%) applied a urethral dose constraint, as specified in PACE-B and RTOG-0938.

**Figure 3:**
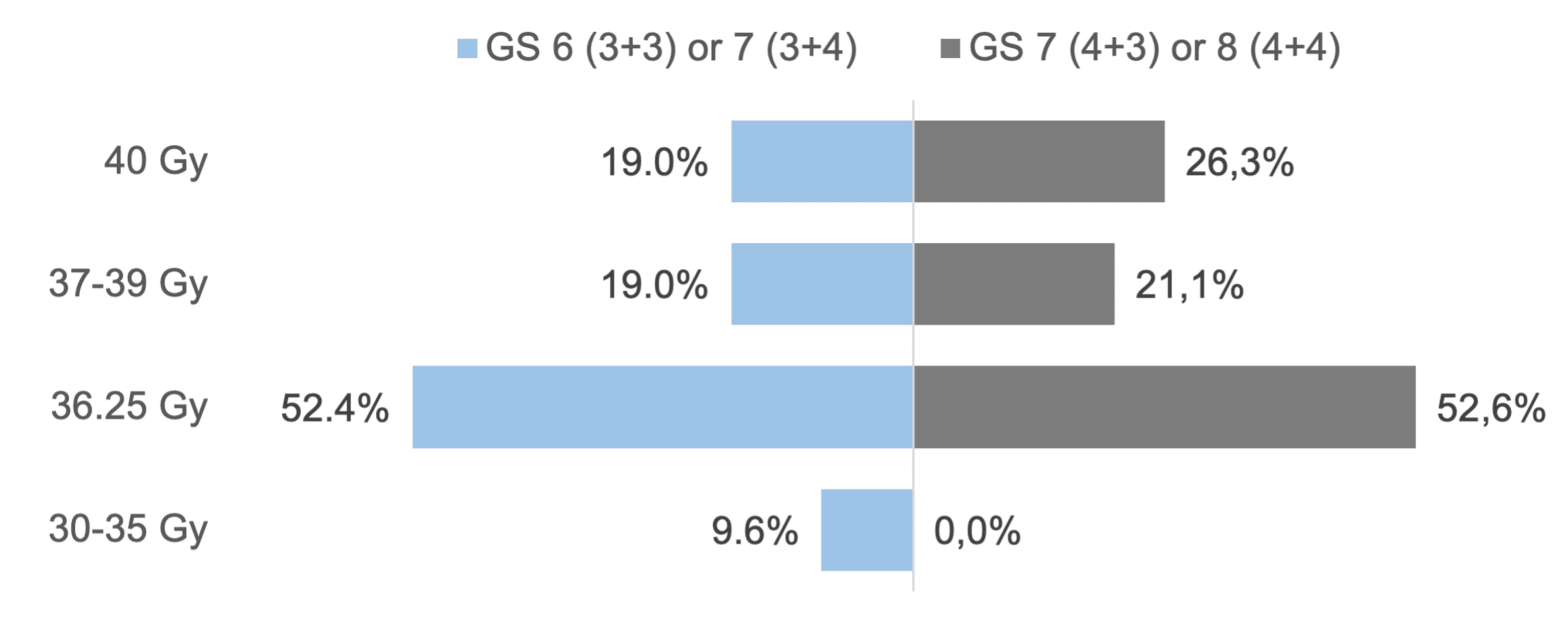
Comparison of the selected dose for patients with Gleason score (GS) 6 (3+3) or 7 (3+4) versus GS 7 (4+3) or 8 (4+4). Image credits: Pablo Castro-Peña, Lourdes Suárez-Villasmil, Patric Kupelian, Miguel Gonzalez, and Cleverson Lopes (generated with Microsoft Excel (Microsoft Corp., USA))

Two clinical scenarios were proposed for patients with Gleason Scores (GS) 6-7 and 7-8, in which each respondent reported the prescribed dose to the prostate CTV in a five-fraction SBRT regimen (Figure 3). The distribution of prescribed CTV doses in both groups showed a greater tendency toward a prescription of 36.25 Gy. Although PACE-B specifies a higher dose to the CTV than to the PTV, 14 (63.6%) of participants prescribed the same dose to both volumes, regardless of the GS.

Concordance between clinical practices and randomized trials

Prior to measuring the concordance of each participant with each of the randomized trials, the nine key variables were classified into two groups. The first group (variables one to five) was defined as “direct factors,” as it refers to radiotherapy prescription parameters that directly affect the treatment plan and its consequent impact on the BED/EQD2 (dose, fractionation). The second group, defined as “indirect factors,” referred to safety, quality, and target delineation procedures, which are related to the accurate and safe delivery of treatment but do not include the prescription itself (Table 3).

**Table 3:** Variables used to determine the percentage of overlap between the randomized trials PACE-B, HYPO-RT-PC, and RTOG-0938 with users of Halcyon equipment who apply SBRT in PCa. SBRT: stereotactic body radiotherapy, PCa: prostate cancer

Variable	Factor type	Aspect
1	Direct	The prescribed dose in patients with a Gleason Score (GS) of 6 (3+3)
2	Direct	The prescribed dose in patients with a GS of 7 (4+3) or 8 (4+4)
3	Direct	The prescribed dose to the PTV (Planning Target Volume) equal to or less than that to the CTV (Clinical Target Volume)
4	Direct	The number of fractions to be administered
5	Direct	The frequency of SBRT administration
6	Indirect	The assignment of a limiting dose to the urethra
7	Indirect	The use of MRI to delineate the prostate volume
8	Indirect	The bladder and rectal preparation of patients through specific diets
9	Indirect	The use of intraprostatic fiducials

**Figure 4:**
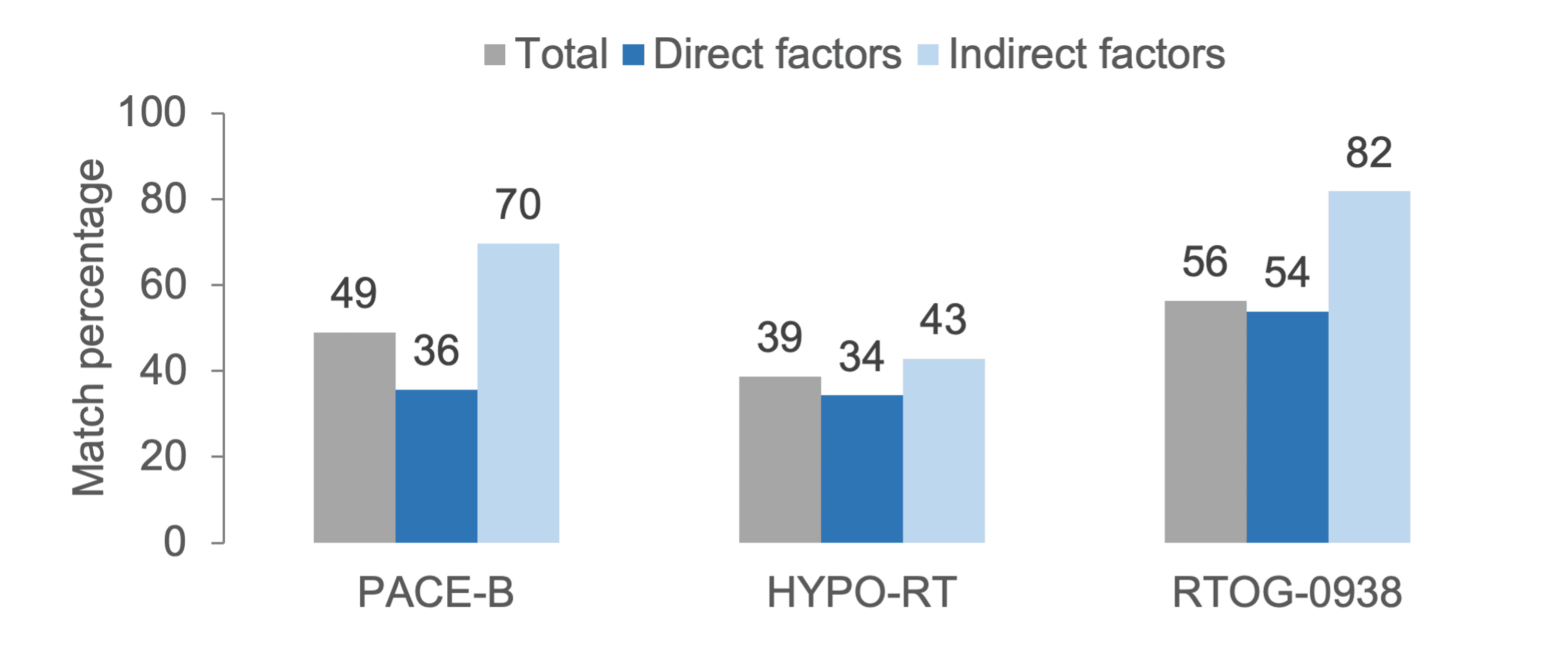
Percentage of concordance between the randomized trials PACE-B, HYPO-RT-PC, and RTOG-0938 and Halcyon equipment users delivering SBRT for prostate cancer (PCa). The percentage was calculated using the nine variables (overall), radiotherapy prescription parameters and treatment planning (direct factors), and safety procedures, quality, and target delineation (indirect factors). A given participant may apply the same characteristic described in more than one randomized trial or may not adhere to any of them; therefore, the percentages do not sum to 100%. SBRT: stereotactic body radiotherapy Image credits: Pablo Castro-Peña, Lourdes Suárez-Villasmil, Patric Kupelian, Miguel Gonzalez, and Cleverson Lopes (generated with Microsoft Excel (Microsoft Corp., USA))

Figure 4 shows the mean concordance of participants with the three randomized trials. Concordance was calculated considering all nine direct and indirect variables (gray bars), as well as separately for the five direct variables (dark blue bars) and the four indirect variables (light blue bars). The graph demonstrates a mean concordance of 36% between participants and the dose prescription aspects (direct factors) established by PACE-B; by contrast, participants showed a 70% concordance in quality control and safety procedures (indirect factors). Overall, the mean concordance of professionals with the three randomized trials was 41.3% for direct factors and 65.0% for indirect factors.

The most notable finding in Figure 4 was the higher concordance between participants and quality assurance and safety procedures (indirect factors) compared with the specific aspects of dose prescription (direct factors).

## Discussion

This study provides a comprehensive overview of the current implementation of prostate SBRT among a group of Latin American professionals using a homogeneous technological platform based on Halcyon^TM^ systems. The results demonstrate a substantial adoption of hypofractionation (86.5%) and SBRT (59.5%), reflecting the global trend toward shorter and more efficient radiotherapy regimens [10,11]. This transition is particularly relevant in LATAM, where the logistical and economic advantages of SBRT, including reduced treatment duration, lower system costs, and improved accessibility for patients facing geographic or financial barriers, may facilitate broader access to treatment.

The analysis included professionals from 10 Latin American countries, with Brazil, Colombia, and Argentina representing the largest proportion of participants. The inclusion of countries with lower representation further supports the regional scope of the study and highlights the diversity of prostate radiotherapy practices across Latin America, even within centers equipped with homogeneous technology.

When the agreement with randomized clinical trials was assessed, a clear pattern of agreement emerged. Adherence was consistently higher for indirect factors related to treatment safety and quality assurance, such as bladder and rectal preparation, use of MRI, and assignment of a urethral dose constraint, while concordance with direct prescription parameters (fractionation and dose prescription) was comparatively lower. This finding is clinically relevant and aligns with the different parameters of the trials used as references in this study.

The high adoption of bladder and rectal preparation protocols, use of magnetic resonance imaging for prostate delineation, and assignment of urethral dose constraints reflect strong alignment with the safety and quality assurance principles emphasized in the trials, particularly in PACE-B [11].

By contrast, lower concordance was observed for direct prescription parameters when compared with the PACE-B trial, which represents the only phase III randomized study evaluating prostate SBRT delivered in five fractions with mature oncological outcomes. Variability in prescribed dose levels and, notably, the frequent practice of prescribing identical doses to the clinical target volume (CTV) and planning target volume (PTV) diverge from the PACE-B protocol, which recommends a higher dose to the CTV relative to the PTV.

This discrepancy suggests that, while safety-related procedures have been widely adopted, there is less consistency in the application of validated prescription strategies that are most directly associated with disease control.

This imbalance between adherence to indirect versus direct factors is particularly relevant given that prescription parameters are central to the therapeutic effectiveness of SBRT.

The findings highlight a gap between the availability of high-level evidence supporting prostate SBRT as a standard treatment option established by the mature oncological outcomes of the PACE-B trial and its uniform implementation in daily clinical practice. Similar concerns regarding the need for consensus in SBRT prescription have been previously reported in the literature and remain especially pertinent in regions lacking region-specific clinical practice guidelines.

Although the present analysis included clinical scenarios of patients across different risk categories, it is important to contextualize that the randomized evidence underpinning the current international adoption of prostate SBRT primarily derives from trials conducted in low- and intermediate-risk populations (PACE-B and RTOG-0938), while HYPO-RT-PC included a broader spectrum but under different clinical conditions. Therefore, the inclusion of high-risk disease scenarios in our survey should not be interpreted as a recommendation or endorsement of SBRT for this subgroup, but rather as a reflection of real-world variation in clinical practice across Latin America. This reinforces the need for structured education, consensus building, and protocol harmonization in the region.

The observed heterogeneity in practice also emphasizes that access to advanced radiotherapy technology alone, although of paramount relevance, is insufficient to guarantee the provision of standardized, high-quality evidence-based treatment. Even among centers using a common technological ecosystem, variability in experience, training, and institutional protocols can significantly influence clinical decision-making. This is particularly relevant in LATAM, where disparities in professional training opportunities and access to continuous education persist [8].

Similar patterns of heterogeneity in SBRT implementation have been reported in other emerging regions, suggesting that these findings are not exclusive to LATAM but reflect a broader challenge in translating evidence into practice across diverse healthcare settings. In this scenario, structured international and regional collaboration platforms offer an opportunity to facilitate knowledge exchange between countries, promote convergence toward standardized protocols, and accelerate learning curves through shared clinical experience.

Halcyon^TM^ technology, which has been validated for SBRT delivery with performance comparable to modern C-arm linear accelerators [19,20], represents one example of technological platforms currently used to support SBRT implementation in diverse healthcare settings.

On the other hand, their integration within structured educational frameworks that reinforce competencies in treatment planning, dosimetric prescription, and quality assurance is of fundamental importance. The results of this study support the need for longitudinal training programs, such as the HUG_CWG SBRT Prostate program implemented by Varian, based on protocols designed to harmonize clinical practice with established international evidence, to maximize the quality of SBRT.

In addition, while urethral dose limitation represents an important safety parameter and was appropriately implemented by most centers, it should be highlighted that, in contemporary prostate SBRT practice, rectal and bladder constraints remain the principal determinants of treatment safety and long-term gastrointestinal and genitourinary outcomes. In this context, the widespread adoption of bladder filling and rectal emptying strategies observed in our cohort is reassuring; however, variability in how consistently these measures are applied throughout the treatment course may affect reproducibility and clinical outcomes. These findings further support the need to strengthen education on standardized constraint implementation and fraction-to-fraction quality assurance within regional SBRT training initiatives.

Among the 63.6% of participants who reported using rectal enemas for prostate SBRT, considerable variability was observed in their application: 13.6% used enemas daily, 40.9% limited their use to computed tomography (CT) simulation, and 9.1% used them only occasionally. As previously reported by Byun et al. [26], the routine use of enema-based rectal preparation for every SBRT fraction, including CT simulation, has been questioned. Nevertheless, performing rectal preparation exclusively at simulation without reproducing it during each SBRT fraction may result in an optimal rectal condition at planning that is not consistently reproducible throughout treatment, potentially affecting treatment accuracy and reproducibility.

In contrast, adherence to urethral dose constraints was high in this cohort. Consistent with recommendations from the PACE-B and RTOG-0938 trials [11,24], 81.8% of participants were assigned a limiting dose to the urethral volume, a practice supported by evidence demonstrating a dose-effect relationship involving the urethra and urinary sphincters in the development of late genitourinary toxicity [27]. Furthermore, previous studies have shown the feasibility of delivering high-dose prostate SBRT while sparing the urethra without compromising tumor coverage [28], supporting an optimal balance between disease control and treatment-related toxicity.

Among the less frequent practices, only 13.6% reported implanting intraprostatic fiducials. Although fiducials are mandatory in HYPO-RT-PC and recommended in PACE-B, the results of the latter trial were not conclusive regarding toxicity or local control [11].

Regarding the use of intraprostatic fiducials, their limited adoption among surveyed participants should not be interpreted as a deficiency in treatment quality. Although fiducials were recommended or required in earlier randomized SBRT trials, they are no longer universally mandatory in modern SBRT workflows, particularly when robust image-guided radiotherapy strategies are available. This is especially relevant within Halcyon-based ecosystems, where daily IGRT solutions allow accurate treatment delivery without compulsory fiducial implantation. Therefore, in this technological context, fiducials should be considered optional rather than a strict quality requirement.

With respect to the need for continuous education, Duma and Moraes [8] note that radiation oncology is not a mandatory component of training programs in LATAM, and most Latin American trainees do not have the opportunity to attend international conferences due to economic constraints. The findings of this study show that 40.5% of participants have not used SBRT, with lack of experience being the main barrier (66.7% of non-users). In addition, a disparity is observed between real-world clinical practice and standardized protocols from randomized trials, particularly in aspects affecting direct prescription parameters (use of non-validated dose prescriptions), as well as inconsistencies in rectal preparation that compromise treatment reproducibility and the limited number of cases treated by several of the surveyed professionals.

The results reported in this study demonstrate heterogeneity in radiotherapy practice, and specifically in prostate SBRT, which may negatively impact SBRT quality (disease control and toxicity), to the detriment of treatment efficacy and patient outcomes. This underscores the strong need to develop and implement easily accessible continuous education programs [18], in which protocolization and standardization of institutional practices are harmonized, and structured data recording with high-quality statistical reporting allows analysis and reporting of LATAM practices at small, medium, and large scales.

In this context, the HUG_CWG framework represents a practical mechanism to address the gaps identified in this study. By bringing together institutions operating within a homogeneous technological ecosystem, HUG_CWG facilitates structured peer-to-peer exchange, dissemination of standardized protocols, and progressive alignment with evidence-based SBRT practices. Beyond technology adoption, this collaborative environment lowers barriers to continuous education, particularly in regions where access to formal training opportunities remains uneven and supports the translation of high-level clinical evidence into routine practice.

The continuous education program developed with the support of Varian Medical Systems [29] is conceived as a comprehensive initiative aimed at improving the quality of prostate SBRT in LATAM through the use of a homogeneous technological platform and standardized clinical protocols within a continuous education framework.

The project promotes a continuous improvement model that combines theoretical and practical education, interactive sessions in virtual environments (Eclipse-ARIA), and direct mentorship by regional experts in prostate SBRT, both remotely and in person. The educational strategy is structured into sequential modules covering planning and dosimetric prescription through quality control and evaluation of clinical outcomes, based on validated international protocols. The virtual learning platform incorporates a continuous education schedule adapted to the learning curve required for proper SBRT implementation, using resources previously validated in the region.

Quality control is performed through periodic evaluations by specialists in the clinical and physical use of integrated platforms (Halcyon-Eclipse-ARIA or equivalents), ensuring treatment accuracy and adherence to established protocols. One of the main indicators to evaluate program effectiveness will be the increase in the average level of participant concordance with parameters defined in published randomized trials, a metric that will allow quantification of the impact of the educational model on the adoption of standardized, evidence-based practices in routine clinical settings.

These findings reinforce a critical concept: access to advanced radiotherapy technology, although essential, is not sufficient to guarantee high-quality and standardized SBRT practice. The persistent variability observed in treatment prescription, dosimetric parameters, and consistency of preparation protocols highlights the fundamental role of structured continuous education, protocol harmonization, and regionally adapted training initiatives to translate technological capability into reproducible clinical quality and patient benefit.

The analysis presented here is descriptive and does not establish causality; therefore, these patterns may be influenced by factors not measured in this study, such as local professional training, resource availability, or protocol adoption by practicing professionals at participating centers. This highlights the need to implement educational strategies and unified protocols to ensure consistent and evidence-based application of prostate SBRT.

Several limitations should be acknowledged. First, the study included a relatively small sample of professionals and institutions, which may not fully represent all prostate SBRT practices across LATAM. Second, the sample was derived from centers using a specific technological ecosystem, which may introduce selection bias. Third, the analysis was based on self-reported practices rather than independent clinical audits or treatment plan review. Finally, the study was designed as a descriptive survey and did not evaluate clinical outcomes, which limits the ability to directly assess the impact of practice variability on patient results.

The findings of this study should therefore be interpreted as a descriptive overview of current clinical practice patterns rather than as evidence of treatment effectiveness or clinical outcomes. Future studies incorporating prospective data collection, treatment plan audits, and patient outcomes will be important to better understand the clinical impact of educational and protocol harmonization initiatives in the region.

## Conclusions

The results of this study demonstrate that prostate SBRT is progressively consolidating in LATAM, with moderate adherence to international randomized clinical trials. However, meaningful variability persists in both direct prescription parameters and indirect quality and safety procedures. This heterogeneity indicates that access to advanced radiotherapy technology, while of utmost importance, is not by itself sufficient to guarantee a standardized, high-quality SBRT practice.

Strengthening structured, sustained, and accessible continuous education, together with regional protocol standardization and robust quality assurance strategies, represents a strategic opportunity to enhance consistency, safety, and clinical benefit. In parallel, fostering a strong culture of structured data recording and high-quality reporting may support continuous quality improvement and generate regional evidence to guide future practice.

Within this framework, collaborative initiatives such as the HUG_CWG provide a scalable and sustainable educational model that facilitates protocol harmonization, quality improvement, and cross-country knowledge exchange, contributing to improved consistency and quality of prostate SBRT delivery in LATAM and other emerging regions, while optimizing the clinical benefit of advanced technological platforms such as Halcyon or equivalent systems.

## Appendices

Study questionnaire

**Table 4:** Questions asked to 37 professionals (radiation oncologists and medical physicists) from 28 institutions in 10 Latin American countries. Source: Pablo Castro-Peña, Lourdes Suárez-Villasmil, Patric Kupelian, Miguel Gonzalez, and Cleverson Lopes

Question #	Item	Response options
1	I provide informed consent for the use of anonymized data and agree to the confidentiality terms.	Yes; No.
2	Professional role	Radiation oncologist; medical physicist.
3	Institution	Open response
4	Country of practice	Open response
5	What radiation therapy technique do you currently use to treat your patients with primary (unoperated) prostate cancer? (single option)	Hypofractionation; SBRT; Normofractionation.
6	Do you perform SBRT Prostate? (single option)	No; I have performed between 5 and 20 patients; I have performed between 21 and 50 patients; I have performed between 51 and 100 patients; I have performed more than 100 patients.
7	In case you have not performed prostate SBRT, what is the reason? (multiple choice)	Yes, prostate SBRT is performed; Implementation in progress (not started); Technical or infrastructure limitations; Lack of experience or knowledge.
8	Do you use magnetic resonance imaging (MRI) to outline (contour) the volume of the prostate? (single option)	I do not perform SBRT; Never; Always; Only to delimit tumor volume (GTV).
9	Do you have a specific bladder and rectal preparation protocol (liquid intake & specific diet) defined for all prostate SBRT patients? (single option)	I do not perform SBRT; Yes; No.
10	Do you use a "diary" rectal enema to perform SBRT? (single option)	I do not perform SBRT; Always; Never; Only for CT simulation, but not during each SBRT fraction; Other.
11	Do you routinely use for SBRT? (multiple choice)	I do not perform SBRT; Intra-prostatic fiducials; Rectal spacer; None of this; Other.
12	In how many fractions do you perform prostate SBRT? (multiple choice)	I do not perform SBRT; 1 fraction; 2 fractions; 3-4 fractions; 5 fractions, 6-10 fractions.
13	How do you prescribe prostate doses? (single option)	I do not perform SBRT; Same dose to PTV as to CTV? (example: PTV and CTV= 36.25 Gy); Lower dose to PTV and higher dose to CTV? (example: PTV = 36.25 Gy and CTV = 40 Gy); Other.
14	Do you perform SBRT prostate on continuous days or every other day? (single option)	I do not perform SBRT; Continuous days; Every other day (one yes, one no).
15	Suppose a patient with Gleason Score 6 (3+3) or 7 (3+4). Regarding the dose of SBRT in the prostate (5 fractions), what dose do you prescribe to the CTV of the prostate? (single option)	I do not perform SBRT; 30 to 35 Gy; 36.25 Gy; 37 to 39 Gy; 40 Gy; more than 40 Gy.
16	Suppose a patient with Gleason Score 7 (4+3) or 8 (4+4). Regarding the dose of SBRT in the prostate (5 fractions), what dose do you prescribe to the CTV of the prostate? (single option)	I do not perform SBRT; 30 to 35 Gy; 36.25 Gy; 37 to 39 Gy; 40 Gy; more than 40 Gy.
17	When you can evidence a tumor nodule by MRI, do you perform a Prostate SIB (that is, a higher dose than that prescribed for the Prostate CTV)? (single option)	I do not perform SBRT; Yes; No.
18	When you prescribe SBRT in the Prostate, do you assign to the urethra a dose limit to be respected (i.e., not to exceed)? (single option)	I do not perform SBRT; Yes; No.
19	When you prescribe SBRT in the Prostate, do you assign the Bladder Trigone a dose limit to be respected (i.e., not to exceed)? (single option)	I do not perform SBRT; Yes; No.
20	In a patient with primary prostate cancer. If indicated: do you perform SBRT in pelvic lymph node areas prophylactically? (single option)	I do not perform SBRT; I do not perform SBRT in pelvic lymph node areas; Yes, I perform a full pelvis; Yes, I perform mini-pelvis; Yes, I perform only involved lymph node(s).
21	If SBRT is performed on pelvic lymph node areas prophylactically, what dose do you prescribe to PTV/CTV? (single option)	I do not perform SBRT; 25 Gy in 5 fractions; > 25 Gy in 5 fractions.
22	Regarding the dose limits for organs at risk (OARs) that you use when performing SBRT, where did you get them from? (multiple choice)	I do not perform SBRT; Unpublished experience; PACE-B, Fase III RCT, Reino Unido/Canadá (Van As et al. 2019; Tree et al. 2022); HYPO-RT-PC, ECA phase III, Denmark (Widmark et al. 2019); MSKCC (Zelefsky et al. 2019); Timmerman Tables (IJROBP 2022); Other.
23	Do you perform prostate SBRT in post-prostatectomy patients?	I do not perform SBRT; Yes; No.
24	Do you do prostate SBRT in patients with post-radiotherapy prostate recurrence?	I do not perform SBRT; Yes; No.
